# Data Mining of the FDA Adverse Event Reporting System and Animal Experiments for Assessment of Rhabdomyolysis Risk Associated with Lipid-lowering Drugs

**DOI:** 10.7150/ijms.109034

**Published:** 2025-02-26

**Authors:** Shinji Kobuchi, Daisuke Sugiyama, Anna Iima, Ami Obuchi, Ayumi Osaka, Ayana Doi, Hikaru Ueta, Satoshi Yokoyama, Kouichi Hosomi, Mitsutaka Takada, Toshiyuki Sakaeda

**Affiliations:** 1 Department of Pharmacokinetics, Kyoto Pharmaceutical University, Kyoto 607-8414, Japan.; 2 Faculty of Pharmacy, Kindai University, 3-4-1, Kowakae, Higashi-osaka, Osaka 577-8502, Japan.

**Keywords:** rhabdomyolysis, cerivastatin, gemfibrozil, FAERS, preclinical pharmacokinetics

## Abstract

Despite extensive research on pharmacokinetic interactions between hydroxymethylglutaryl-CoA reductase inhibitors (statins) and fibrates, the underlying pharmacodynamic mechanisms contributing to the increased risk of rhabdomyolysis remain unclear. This study aimed to determine the differences among statins or fibrates in terms of their susceptibility to rhabdomyolysis. The data mining of FDA Adverse Event Reporting System (FAERS) suggested the association of both statins and fibrates with rhabdomyolysis and the add-on effect of their combinations. In rats, their administration was associated with outliers in creatine phosphokinase and myoglobin levels and a larger distribution of data than in the control. Additionally, co-administration of cerivastatin increased the gemfibrozil concentration in skin and muscle tissues by more than two-fold without an increase in systemic exposure to gemfibrozil, suggesting that an alteration in the pharmacokinetics of gemfibrozil might contribute to an increased risk of rhabdomyolysis when cerivastatin and gemfibrozil are co-administered. Taken together, caution is uniformly needed in combination therapy with statins and fibrates because of the increased risk of rhabdomyolysis.

## Introduction

Hydroxymethylglutaryl-CoA reductase inhibitors, known as statins, are recommended as the first class of lipid-lowering drugs for the primary and secondary prevention of cardiovascular events 1, 2. Currently, lovastatin, simvastatin, pravastatin, fluvastatin, atorvastatin, rosuvastatin, and pitavastatin are available worldwide for this purpose. High-intensity treatment with statins reduces low-density lipoprotein cholesterol (LDL-C) by more than 50% and triglycerides by up to 40% 1, 2. Fibrates, such as fenofibrate, bezafibrate, and gemfibrozil, are also used for dyslipidaemia, which exert their effects via activation of the peroxisome proliferator receptor alpha (PPAR-α). Treatment with fibrates results in the reduction of LDL-C by 20% and triglycerides by approximately 50% 1, 2. Fibrates have been prescribed for a long time prior to the introduction of statins; however, recent studies have shown a higher incidence of adverse events than those recorded with statins, except for myalgia 3.

In 2001, cerivastatin was withdrawn from the global market owing to drug-related fatal rhabdomyolysis 4, 5. Reviewing spontaneous reports in the FDA Adverse Event Reporting System (FAERS) showed that mortality rates were 16-86-fold higher than those reported with other statins 4, 5. Additionally, gemfibrozil was co-administered in 12 of 31 reported deaths among cerivastatin users 4. Hence, their interaction has attracted interest. Immediately thereafter, it was found that gemfibrozil increased the exposure to cerivastatin exposure by more than five-fold 6, which encouraged a focus on basic pharmacokinetic and/or clinical investigations rather than pharmacodynamic investigations. Reports accumulated in the past decades have been well organized, resulting in a management strategy for the pharmacokinetic drug interactions of statins 7, 8. However, the underlying mechanisms remain unclear, especially from a pharmacodynamic perspective, and at present, a consensus warning has been issued for the combination therapy of statins and fibrates due to the potential increase in the risk of rhabdomyolysis.

This study aimed to determine the differences among statins or fibrates in terms of susceptibility to rhabdomyolysis. The FAERS database was re-reviewed using reports published after cerivastatin withdrawal. Big data mining results are affected by various factors 9, and the association with rhabdomyolysis has been analysed as a signal for each statin or fibrate 10. Furthermore, the study also aimed to evaluate the add-on effect of fibrates with each statin, and the effect of statins was analysed for each fibrate. To support these data, the markers of muscle disorder, creatine phosphokinase (CK) and myoglobin levels in plasma, and distribution into organs/tissues were evaluated using rats after the administration of cerivastatin, gemfibrozil, and both. In the animal experiments, pravastatin and fenofibrate were used as controls and, which were selected based on the results of the FAERS database analysis.

## Materials and methods

### Data mining of the FAERS database

The data were obtained from the public release of the FAERS database, which covers the period from the first quarter of 2004 to the end of 2016. The data structure adheres to the international safety reporting guideline, ICH E2B, and a report consists of 7 data tables: patient demographics and administrative information (DEMO), drug/biologic information (DRUG), adverse events (REAC), patient outcomes (OUTC), report sources (RPSR), drug therapy start and end dates (THER), and indications for use/diagnosis (INDI). The adverse events in REAC are coded using preferred terms in the Medical Dictionary for Regulatory Activities (MedDRA) terminology. Here, MedDRA/J version 21.1J was used.

In this study, eight statins (atorvastatin, cerivastatin, fluvastatin, lovastatin, pitavastatin, pravastatin, rosuvastatin, and simvastatin) and five fibrates (bezafibrate, clinofibrate, clofibrate, fenofibrate, and gemfibrozil) were selected. Prior to analysis, all drug names were unified into generic names, and spelling errors were corrected. For adverse events, rhabdomyolysis was selected using the Standardized MedDRA Query (SMQ) of code 20000002. Prior to data mining, duplicate reports were deleted according to the FDA's recommendation to adopt the most recent CASE number, resulting in a reduction in the number of reports from 8,867,135 to 7,343,647.

A two-by-two contingency table was created for each stain or fibrate, and its association with rhabdomyolysis was analysed. For the add-on analysis, reports on statins were extracted, and the association was compared with and without any fibrates. Similarly, the add-on effect of statins was analysed for each fibrate. The reporting odds ratio (ROR) and the information component (IC) were used for the data-mining algorithms. For ROR, a signal was detected if the lower limit of the 95% two-sided confidence interval exceeded 1 11. Signal detection using IC is done using the IC025 metric, a lower limit of the 95% two-sided confidence interval of the IC, and a signal was detected if the IC025 value exceeded 0 12.

### Chemicals and animals

Gemfibrozil and fenofibrate were purchased from the Tokyo Chemical Industry Co., Ltd. (Tokyo, Japan). Cerivastatin was purchased from Sigma-Aldrich Co. St. Louis, MO). Pravastatin was supplied by Wako Pure Chemical Industries, Ltd. (Osaka, Japan).

Ten-week-old male Wistar rats were obtained from Nippon SLC Co. Ltd. (Japan). The rats were acclimated to a temperature-controlled room under a 12-h light/ 12-h dark cycle with ad libitum access to food and water during the experimental period. The animal experimental procedures were approved by the Animal Experimentation Ethics Committee of Kyoto Pharmaceutical University and conducted in accordance with the Kyoto Pharmaceutical University Guidelines for Animal Experimentation.

### Creatine phosphokinase (CK) and myoglobin levels in rats treated with statins, fibrates, or their co-administrations

The effects of co-administration of cerivastatin and gemfibrozil on CK and myoglobin levels in plasma were evaluated in rats after oral administration. The effects of the co-administration of pravastatin and fenofibrate were also investigated. The rats were divided into seven groups: vehicle (propylene glycol) control (n = 15), gemfibrozil, cerivastatin, gemfibrozil plus cerivastatin, fenofibrate, pravastatin, and fenofibrate plus pravastatin (n = 18 in each group). The dose of each drug was determined based on the clinical dose for patients of 50 kg body weight (i.e. 5 mg clinical dose for patients was considered as 0.1 mg/kg for administration to rats): 25 mg/kg gemfibrozil, 16 μg/kg cerivastatin, 5 mg/kg fenofibrate, and 0.4 mg/kg pravastatin. Each drug solution was prepared by dissolving each drug in propylene glycol. To normalize the effects of propylene glycol on CK and myoglobin levels, the dosing volume was set at 2 mL/kg in all rats. Under isoflurane anesthesia, blood samples (1.5 mL) were collected from the external left jugular vein 8 h after administration into heparinized centrifuge tubes and centrifuged immediately at 14,000×*g* for 15 min. To minimize the potential risk of esophageal contraction and aspiration under isoflurane anesthesia, the drug was administered by using a long gavage tube (12 cm) to ensure direct gastric delivery, preventing reflux into the oral or nasal cavity. The absence of regurgitation, gagging, or any signs of aspiration during and after administration was visually confirmed. The sampling time point was selected based on the time course of CK and myoglobin levels following muscle injury, as both markers peak 6-12 hours after the injury. The CK and myoglobin levels in plasma were determined at a commercial laboratory (Kyoto Biken Laboratories Inc., Kyoto, Japan).

### Pharmacokinetic study of the combination of gemfibrozil with cerivastatin and fenofibrate with pravastatin in rats

The effects of co-administration with cerivastatin on the pharmacokinetics of gemfibrozil were evaluated in rats after oral administration. The effects of co-administration of pravastatin on fenofibrate pharmacokinetics were also measured; however, in this case, the levels of fenofibric acid (an active metabolite of fenofibrate) were evaluated. The rats were divided into four groups: gemfibrozil (n = 7), gemfibrozil plus cerivastatin (n = 7), fenofibrate (n = 10), and fenofibrate plus pravastatin (n = 10). The sample size was determined based on the preliminary experiments. The dosage and preparation of the drug solutions are described above. Blood samples (250 μL) were withdrawn from the external left jugular vein at 0.5, 1, 1.5, 2, 3, 4, 6, and 8 h after administration, collected into heparinized centrifuge tubes, and then centrifuged immediately at 14,000×*g* for 15 min. The obtained plasma samples were stored at -80 °C until liquid chromatography-tandem mass spectrometry (LC-MS/MS) analysis.

The distribution of gemfibrozil and fenofibric acid in the liver, kidney, skin, and muscle tissues was measured. At 8 h after administration, the rats were immediately euthanized by exsanguination, transcardially perfused with phosphate-buffered saline (PBS, pH 7.4), and the organs/tissues were collected. The rationale for selecting this time point is based on the pharmacokinetic profiles of fibrates and statins, where their plasma concentrations peak within 1-4 hours, followed by tissue distribution and subsequent elimination. Skin samples were collected from the depilated abdominal region (approximately 1 g), and muscle samples were obtained from the quadriceps femoris of the hind limb (approximately 2 g). Organs and tissues were homogenized in PBS (nine-fold volume of each sample weight) using a homogenizer (PT 10-35 GT; Kinematica AG, Lucerne, Switzerland). The homogenate sample was centrifugated at 3000×*g* for 15 min, and resultant supernatant fractions were stored at -80 °C until LC-MS/MS analysis.

### LC-MS/MS assay

The concentrations of gemfibrozil and fenofibric acid in the plasma and organ/tissues were determined by LC-MS/MS, as previously reported 13, 14 with minor modifications. The LC-MS/MS system comprised an API 3200 triple-quadrupole mass spectrometer (Sciex, Framingham, MA, USA). The flow rate of the mobile phase was 0.2 mL/min, and chromatographic separations were conducted using a Quicksorb ODS (2.1 × 150 mm, 5 μm size; Chemco Scientific Co., Ltd., Osaka, Japan), maintained at 50°C. The mass spectrometer used a selected reaction monitoring method with 249.0 → 121.0 for gemfibrozil in positive ion mode and 317.0 → 231.0 for fenofibric acid in negative ion mode. The assay was conducted using simplified protein precipitation and liquid-liquid extraction. Briefly, acetonitrile (100 μL) was added to a 100-μL aliquot of plasma or organ/tissue samples for deproteinization. After vigorous mixing for 30 s, the mixture was centrifuged at 14,000 *g* for 15 min. The supernatant was transferred to a clean 2.0-mL centrifuge tube, and 0.1% formic acid (100 μL) and ethyl acetate/diethyl ether (1.0 mL; 1:1, v/v) was added. After the tube was vortexed for over 30 s and centrifuged at 14,000 × g for 15 min, the organic layer was transferred to a clean 1.5-mL centrifuge tube and evaporated to dryness under a stream of nitrogen at 60°C. The residue was reconstituted with the mobile phase for each analyte as follows: gemfibrozil, 0.1% formic acid/acetonitrile (1:1, v/v), and fenofibric acid, 0.1% formic acid/acetonitrile (1:4, v/v). The reconstituted solution (30 µL) was injected into the LC-MS/MS system. The lower limit of quantification for each analyte was <0.01 μg/ml from 100 μl of samples. Each calibration curve was linear over the lower limit of quantification, with a correlation coefficient >0.99.

### Statistical analysis

The data for CK and myoglobin levels are depicted using a box plot with a lower quartile (Q1), median (Q2), upper quartile (Q3), whisker (Q1 - 1.5 interquartile range [IQR] and Q3 + 1.5 IQR), and outliers representing > 1.5 IQR. Data from multiple groups were compared using one-way analysis of variance (ANOVA), followed by Bonferroni adjustment. The data of pharmacokinetic experiments are described as the mean ± standard deviation (S.D.). Statistical differences were evaluated using the Student's unpaired t-test. Differences between means were considered statistically significant at p < 0.05.

## Results

### Data mining of the FAERS database

Table 1 summarizes the ROR and IC values with 95% CI for the eight statins and three fibrates. Clinofibrate and clofibrate were also analysed; however, reliable values could not be obtained owing to the small number of reports. Signals were detected for eight statins and three fibrates, indicating their association with rhabdomyolysis. The signalling scores were highest for cerivastatin (ROR: 42.38 [95% CI: 31.10-57.75], IC: 4.39 [95% CI: 3.95-4.83]) and lowest for pravastatin (ROR: 3.84 [95% CI: 3.54-4.16], IC: 1.89 [95% CI: 1.78-2.01]) among statins, and highest for gemfibrozil (ROR: 18.01 [95% CI: 16.56-19.59], IC: 4.02 [95% CI: 3.90-4.14]) and lowest for fenofibrate (ROR: 5.78 [95% CI: 5.34-6.26], IC: 2.47 [95% CI: 2.35-2.58]) among fibrates. Table 2 shows the results of the add-on of fibrates to each of the eight statins. The association with rhabdomyolysis was compared between the patients who were administered fibrates and those who were not. Signals were detected for all statins except pitavastatin. Table 3 shows the values for three fibrates. Signals were detected with all the fibrates.

### CK and myoglobin levels in rats treated with fibrate, statin, or their co-administrations

**Figure 1:**
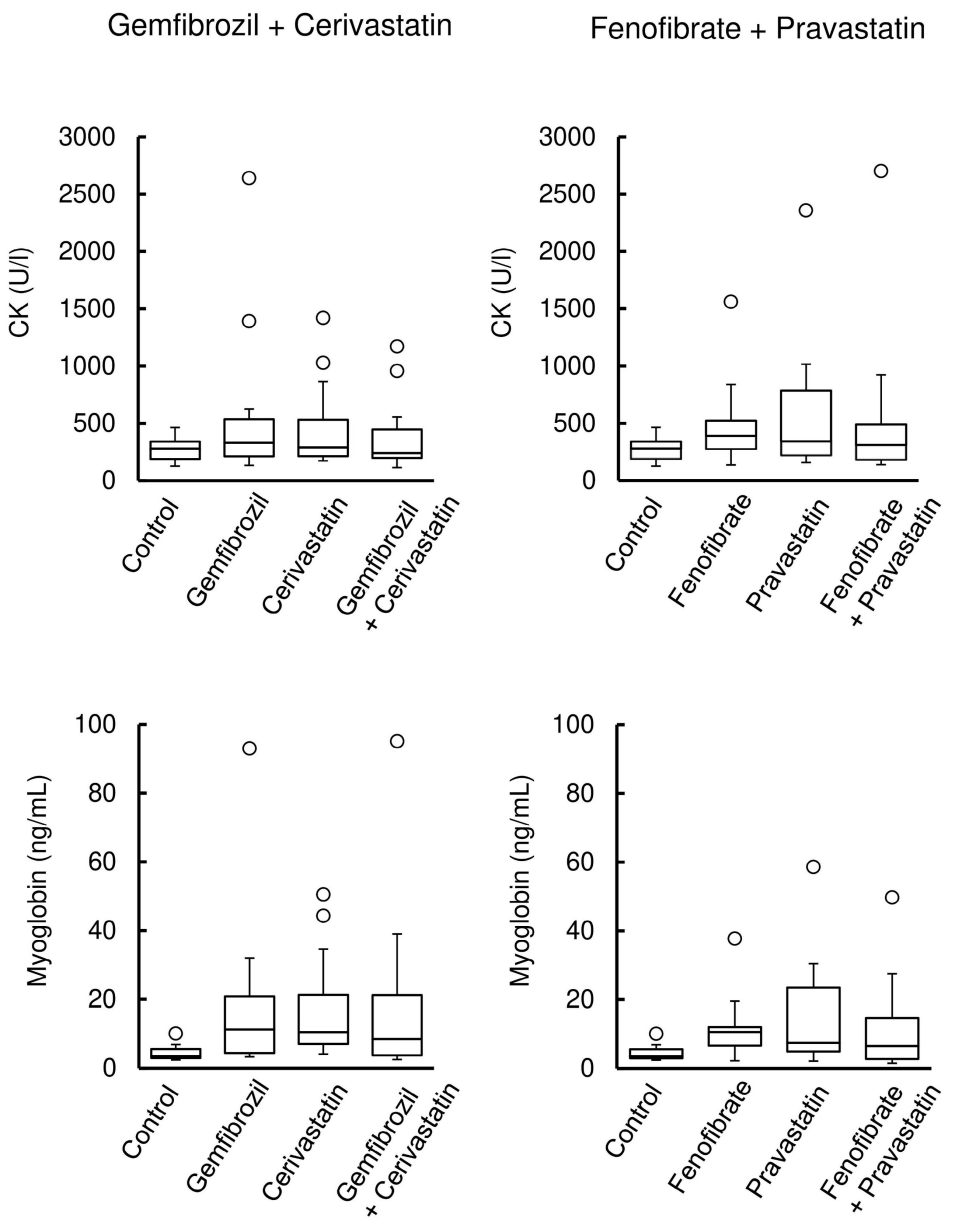
Creatine kinase (CK) and myoglobin levels in rats treated with fibrate, statin, or their co-administrations. Box plots display the medians, interquartile range (IQR) with whiskers extending the range from minimum (25th percentile minus 1.5 × IQR) to maximum (75th percentile plus 1.5 × IQR), and outliers >1.5 × IQR (n = 15 in the control group; n = 18 in the drug treatment group).

Figure 1 (left) shows the CK and myoglobin levels in rats after the administration of cerivastatin, gemfibrozil, and their co-administration, whereas those after pravastatin, fenofibrate, and their co-administration are shown in Figure 1 (right). Their administration was associated with the observation of outliers in CK and myoglobin levels and a larger distribution of data than in the control. However, no significant differences were observed between the groups.

### Pharmacokinetics of gemfibrozil with cerivastatin and fenofibrate with pravastatin in rats

**Figure 2:**
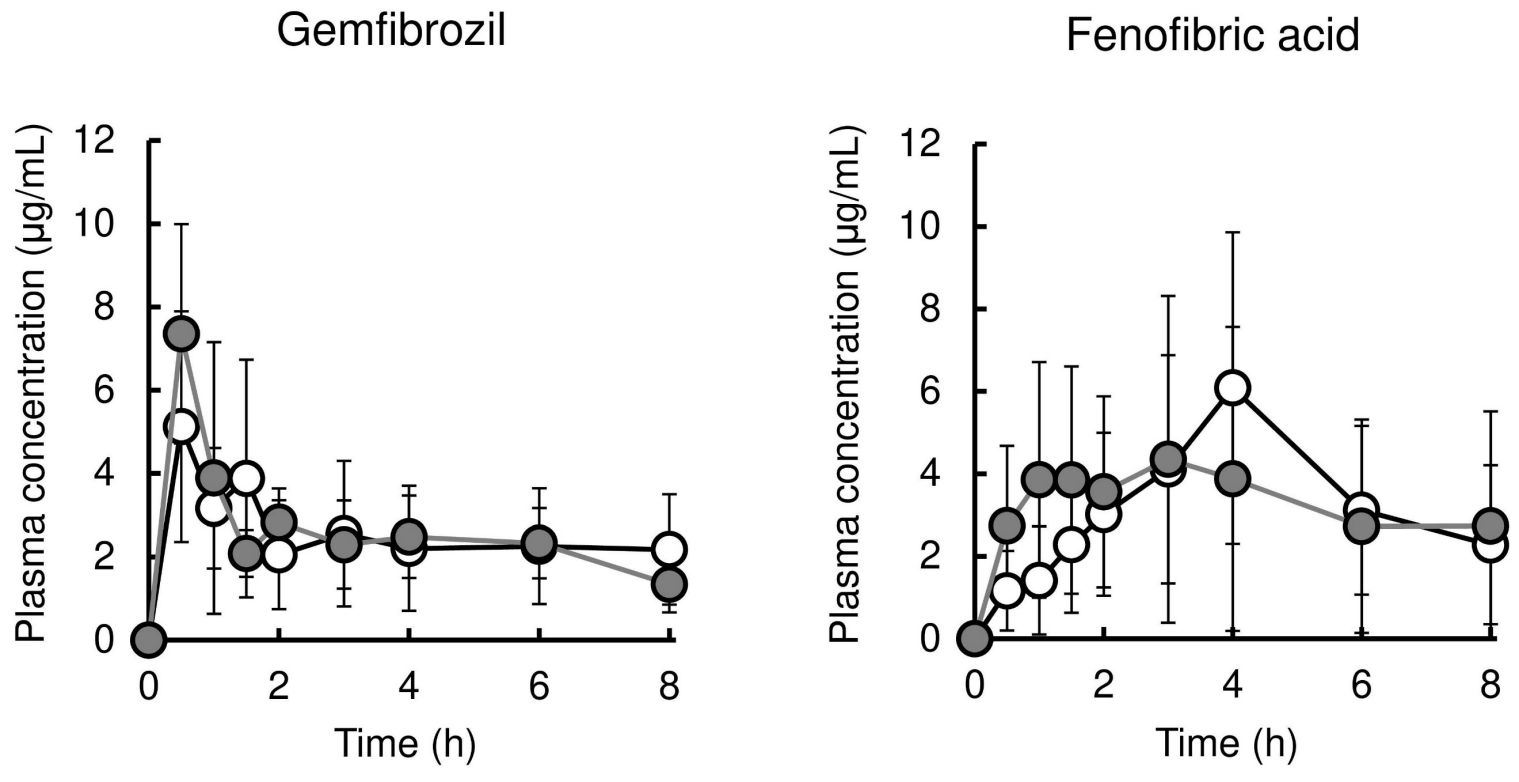
Mean plasma concentrations of fibrate in rats after administration of fibrate or co-administration with statin. The left panel shows plasma gemfibrozil levels in rats after oral administration of gemfibrozil (25 mg/kg) or co-administration with cerivastatin (0.016 mg/kg). The right panel shows plasma fenofibric acid levels in rats after oral administration of fenofibrate (5 mg/kg) or co-administration with pravastatin (0.4 mg/kg). Results are presented as the mean ± S.D. (n = 5-10). White and Gray circles represent plasma concentrations in rats treated with fibrate alone or fibrate plus statins, respectively.

Figure 2 (left and right, respectively) shows the time profile of plasma concentration of gemfibrozil with or without cerivastatin and that of fenofibric acid with or without pravastatin. The pharmacokinetic parameters are listed in Table 4. Co-administration with cerivastatin had no effect on the pharmacokinetics of gemfibrozil. Pravastatin co-administration also did not result in significant alteration of pharmacokinetics of fenofibric acid, except for the reduction of T_max_ value from 3.9±1.6 h to 1.7±1.0 h.

Table 5 summarizes the concentrations of gemfibrozil and fenofibric acid in the plasma, liver, kidneys, skin tissue, and muscle tissue of rats with or without cerivastatin or pravastatin. Co-administration with cerivastatin increased gemfibrozil concentrations in the skin tissue (from 0.23±0.03 to 1.2±0.5 μg/g of tissues) and muscle tissue (from 0.09±0.03 to 0.21±0.08 μg/g of tissues); however, co-administration with pravastatin had no effects on fenofibric acid concentrations in the organ/tissues.

## Discussion

The current analysis of the FAERS database suggested an association of both statins and fibrates with rhabdomyolysis and the add-on effect of their combinations (Tables 1, 2, and 3). This was a retrospective observational study based on spontaneous reports that only provided a signal, which was defined as the reported information on a possible causal relationship between an adverse event and a drug, with the relationship being unknown or incompletely documented previously 10. The results of the analysis of the FAERS database depend on the report quality, time window, data mining algorithms, and other factors 9. The number of reports containing cerivastatin decreased in a time-dependent manner after cerivastatin withdrawal, with a decrease in signalling scores, that is, ROR and IC values, in our analysis (data not shown). Thus, the results must be validated using well-organized, large-scale observational, or prospective intervention studies. Graham *et al.* 15 indicated that the risk of rhabdomyolysis was similar and low for monotherapy with atorvastatin, pravastatin, and simvastatin, and a combination with fibrate increased the risk. Engar *et al.* 16 also showed a lower risk of rhabdomyolysis for monotherapy with statins or fibrates than for combination therapy. In contrast, the ACCORD study, a randomized controlled trial with 5,518 patients with type 2 diabetes mellitus, showed that the combination with fenofibrate indicated no additional risk of muscle damage, not of rhabdomyolysis, compared to monotherapy with simvastatin 17. Taken together, the rhabdomyolysis risk of statin-fibrate combination therapy might vary according to the statin and fibrate used and be sometimes similar to that observed with monotherapy; however, the data available suggest that combination therapy may be inferior to monotherapy in terms of safety.

Outliers in CK and myoglobin levels were found. Their variations increased after the administration of cerivastatin or gemfibrozil in rats, but the marker levels showed no further alteration in their co-administration (Figure 1). As controls, pravastatin and fenofibrate were selected based on the signalling scores obtained by analysis of the FAERS database; however, no significant difference was observed among these groups (Figure 1). CK and myoglobin leak into the blood in the cases of skeletal muscle fibre damage and are widely used as biomarkers for monitoring muscle injury. Rhabdomyolysis is a rare adverse event in humans 4, 5, 15, 16. Although our results suggest that statins and fibrates cause muscle injury, the lack of a clear difference between monotherapy and combination therapy in CK and myoglobin levels may be attributed to several factors. First, muscle injury may have already occurred to some extent with monotherapy, making the additional impact of combination therapy less pronounced. This is consistent with clinical findings where combination therapy does not always lead to a significant increase in muscle toxicity markers. Second, the effects of fibrate-statin combinations on muscle toxicity may vary depending on the specific agents used, as suggested by the observed differences between the cerivastatin-gemfibrozil and fenofibrate-pravastatin groups. Additionally, inter-individual variability in drug response may have contributed to the observed outliers.

Cerivastatin is actively transported into the liver by the organic anion-transporting polypeptide 1B and is then metabolized by CYP3A4 and CYP2C8 18-20. Increased susceptibility to rhabdomyolysis upon co-administration of gemfibrozil might be attributed to an increase in the systemic exposure to cerivastatin via both transporter- and P450-mediated inhibition in the liver 21. In this study, co-administration of cerivastatin increased gemfibrozil concentration in the skin and muscle tissues by more than two-fold, although it had no effect on systemic exposure to gemfibrozil (Tables 4 and 5). Since these tissues account for a small fraction of overall drug distribution and their concentrations remain relatively low compared to plasma and liver, the observed increase is unlikely to significantly impact systemic exposure. Little information is available concerning the molecular mechanisms defining the distribution of gemfibrozil in these tissues; however, an alteration in the pharmacokinetics of gemfibrozil might contribute to an increased risk of rhabdomyolysis when cerivastatin and gemfibrozil are co-administered.

Although interspecies differences in drug disposition and myotoxicity are well known, previous studies have demonstrated that the rat model is useful for investigating drug-induced myopathy, including muscle injury caused by statins and fibrates 22, 23. While pharmacokinetic profiles of these drugs differ between species, the rat model remains valuable for understanding the basic mechanisms of muscle injury, providing insights that can inform human studies, where these drugs may cause more significant myotoxic effects in some individuals. Therefore, despite species differences, the rat model offers valuable pharmacological and toxicological insights.

The current study had some limitations. First, the FAERS database provides only signals, and the association between cerivastatin or gemfibrozil and rhabdomyolysis must be validated. Second, alterations in CK and myoglobin levels were found in rats after the administration of cerivastatin or gemfibrozil; however, there is little information on the relationship between the elevation of these biomarkers and rhabdomyolysis. Third, the effect of statins on the pharmacokinetics of fibrates at clinically relevant doses was investigated, while the reverse interaction was not assessed due to statin concentrations falling below the quantification limit. Although higher doses enabled detection, they did not reflect clinical conditions. Further studies are required to assess bidirectional pharmacokinetic interactions. Finally, this study employed a single-dose administration of statins and fibrates in rats, which may not fully replicate the clinical situation where adverse events like rhabdomyolysis are typically observed after repeated administration. Since rhabdomyolysis is often triggered by acute muscle injury, it was hypothesized that a single-dose administration would be sufficient to detect changes in biomarkers such as CK and myoglobin. Although significant changes in biomarkers were not observed, outliers in CK and myoglobin levels were noted, along with alterations in the tissue distribution of gemfibrozil, especially in muscle and skin. These findings provide important insights into the mechanism of tissue-specific distribution changes, even with a single dose. However, the possibility remains that repeated administration could yield different pharmacokinetic or pharmacodynamic effects. Further studies with repeated dosing are warranted to better understand the long-term impact of co-administration of statins and fibrates on rhabdomyolysis risk.

## Conclusions

Analysis of the FAERS database suggested associations of both statins and fibrates with rhabdomyolysis and the add-on effect of their combinations. Outliers in CK and myoglobin levels were found, and their variations increased after the administration of cerivastatin or gemfibrozil in rats. Gemfibrozil concentrations in the skin and muscle tissues were increased by the co-administration of cerivastatin, which might contribute to an increased risk of rhabdomyolysis when cerivastatin and gemfibrozil are co-administered.

## Figures and Tables

**Table 1:** Association between statins, fibrates, and rhabdomyolysis

Drug	ROR (95%CI)	IC (95% CI)
		
atorvastatin	5.11 (4.92, 5.30)	2.17 (2.11, 2.22)
cerivastatin	42.38 (31.10, 57.75)	4.39 (3.95, 4.83)
fluvastatin	13.70 (12.15, 15.46)	3.64 (3.47, 3.81)
lovastatin	5.02 (4.47, 5.63)	2.27 (2.11, 2.44)
pitavastatin	9.24 (7.72, 11.06)	3.07 (2.81, 3.33)
pravastatin	3.84 (3.54, 4.16)	1.89 (1.78, 2.01)
rosuvastatin	7.05 (6.72, 7.39)	2.69 (2.62, 2.75)
simvastatin	12.27 (11.91, 12.64)	3.23 (3.19, 3.27)
		
bezafibrate	12.94 (10.21, 16.39)	3.42 (3.08, 3.76)
fenofibrate	5.78 (5.34, 6.26)	2.47 (2.35, 2.58)
gemfibrozil	18.01 (16.56, 19.59)	4.02 (3.90, 4.14)
		

ROR: reporting odds ratio, IC: information component

**Table 2:** Effects of add-on fibrates on statin signaling during rhabdomyolysis

Drug	ROR (95%CI)	IC (95% CI)
		
atorvastatin	1.80 (1.54, 2.10)	0.79 (0.57, 1.01)
cerivastatin	8.07 (4.15, 15.72)	1.45 (0.72, 2.18)
fluvastatin	1.80 (1.11, 2.93)	0.70 (0.03, 1.38)
lovastatin	2.18 (1.51, 3.13)	0.96 (0.45, 1.47)
pitavastatin	1.43 (0.62, 3.30)	0.38 (-0.74, 1.50)
pravastatin	2.34 (1.81, 3.02)	1.08 (0.72, 1.45)
rosuvastatin	2.05 (1.74, 2.41)	0.93 (0.70, 1.16)
simvastatin	2.48 (2.25, 2.73)	1.17 (1.03, 1.31)
		

ROR: reporting odds ratio, IC: information component

**Table 3:** Effects of add-on statins on fibrate signals in rhabdomyolysis

Drug	ROR (95%CI)	IC (95% CI)
		
bezafibrate	2.35 (1.46, 3.77)	0.67 (0.10, 1.25)
fenofibrate	1.48 (1.27, 1.74)	0.29 (0.09, 0.48)
gemfibrozil	9.97 (7.79, 12.77)	1.00 (0.83, 1.16)
		

ROR: reporting odds ratio, IC: information component

**Table 4:** Pharmacokinetic parameters of gemfibrozil and fenofibric acid in rats administered with or without statins.

Parameters	Gemfibrozil						Fenofibric acid					
	Mono			+ Cerivastatin			Mono			+ Pravastatin		
t_1/2_ (h)	4.0	±	0.8	4.5	±	1.7	3.1	±	1.8	4.4	±	3.5
T_max_ (h)	1.2	±	0.9	0.6	±	0.2	3.9	±	1.6	1.7	±	1.0*
C_max_ (μg/mL)	5.9	±	2.8	7.6	±	2.7	6.3	±	3.5	6.1	±	3.4
Vd/F (L/kg)	8.1	±	7.2	5.5	±	1.9	1.0	±	1.1	1.1	±	0.7
CL_tot_/F (L/h/kg)	1.4	±	1.4	0.9	±	0.4	0.24	±	0.22	0.26	±	0.21
AUC_0-∞_ (μg∙h/mL)	26.4	±	12.2	30.5	±	9.2	36.5	±	25.9	46.0	±	43.7

These data were obtained 8 h after drug administrations. Each value represents the mean ± S.D. (n=5-10). Statistical significance against Mono was evaluated by unpaired Student's t-test. *p < 0.05 statistically significant difference vs. the Mono data.

**Table 5:** Plasma and organ/tissue concentrations of gemfibrozil and fenofibric acid in rats with and without statin co-administration.

Parameters	Gemfibrozil						Fenofibric acid					
	Mono			+ Cerivastatin			Mono			+ Pravastatin		
Plasma	2.9	±	0.6	3.1	±	2.9	7.0	±	4.2	9.2	±	3.9
Liver	24.0	±	10.0	31.4	±	12.4	12.2	±	5.3	13.5	±	4.7
Kidneys	1.2	±	0.9	1.6	±	0.2	3.9	±	2.3	3.6	±	2.1
Skin	0.23	±	0.03	1.2	±	0.5*	1.5	±	0.7	1.1	±	0.5
Muscle	0.09	±	0.03	0.21	±	0.08*	0.21	±	0.11	0.22	±	0.07

These data were obtained 8 h after drug administrations. Each value represents the mean ± S.D. (μg/mL or g of tissues, n=5-8). Statistical significance against Mono was evaluated by unpaired Student's t-test. *p < 0.05 statistically significant difference vs. the Mono data.
